# Glial cells and neurologic autoimmune disorders

**DOI:** 10.3389/fncel.2022.1028653

**Published:** 2022-10-26

**Authors:** Zhao-Qing Li, Tong-Xin Li, Miao Tian, Ze-Sheng Ren, Chen-Yi Yuan, Rui-Kun Yang, Su-Juan Shi, Hui Li, Zhen-Zhen Kou

**Affiliations:** Department of Anatomy, Histology and Embryology, K. K. Leung Brain Research Centre, The Fourth Military Medical University, Xi’an, China

**Keywords:** neurologic autoimmune disorders, glial cells, autoantibody, multiple sclerosis, Guillain–Barre syndrome, neuromyelitis optica spectrum disorders, myelin oligodendrocyte glycoprotein antibody-related diseases

## Abstract

Neurologic autoimmune disorders affect people’s physical and mental health seriously. Glial cells, as an important part of the nervous system, play a vital role in the occurrence of neurologic autoimmune disorders. Glial cells can be hyperactivated in the presence of autoantibodies or pathological changes, to influence neurologic autoimmune disorders. This review is mainly focused on the roles of glial cells in neurologic autoimmune disorders and the influence of autoantibodies produced by autoimmune disorders on glial cells. We speculate that the possibility of glial cells might be a novel way for the investigation and therapy of neurologic autoimmune disorders.

## Introduction

Neurologic autoimmune disorders are caused by an adaptive immune response directed against an antigen expressed within the nervous system, mainly involving young and middle-aged people with high mortality and disability (Rubin et al., 2018; Bhagavati, 2021). It is reported that environmental genetic susceptibility and various stress factors, including virus infection, childbirth, overwork, trauma, emotional agitation, and vaccination contribute to the development of neurologic autoimmune disorders (Fujinami, 2001). To date, there are more than 30 known autoimmune diseases of the nervous system. The most common of them are multiple sclerosis (MS) and Guillain–Barre syndrome (GBS).

It has been known that the etiology of these neurologic autoimmune disorders might have three potential mechanisms: (1) the common autoimmune response to myelin antigens or epitopes in the central nervous system (CNS) and peripheral nervous system (PNS); (2) the high general susceptibility to autoimmune diseases, for instance, autoimmune diseases may be caused or exacerbated by immunomodulatory therapy; and (3) the co-occurrence in both CNS and PNS could be a coincidence (Kamm and Zettl, 2012). Almost every structure of the CNS or PNS may become the target of autoimmune attacks. However, the exact causes of the neurologic autoimmune disorders remain unclear.

Glial cells play a key role in brain physiology, metabolism, development, and even nervous system diseases (Herculano-Houzel, 2014). Glial cells are involved in neuroinflammation and synaptic homeostasis, which are important for maintaining the physiological function of the nervous system. Recently, great attention has been paid to glial cells in nervous system diseases, especially neurologic autoimmune disorders (Brambilla, 2019; Voet et al., 2019). Myelination of axons is formed by oligodendrocytes and Schwann cells, which form an electrical insulator capable of rapid signal transmission. The importance of oligodendrocytes in the pathology of demyelinating diseases such as MS has been recognized, because myelin loss directly affects nerve transmission. Moreover, oligodendrocyte regulates neuronal metabolic support, which attributes to neuronal existence or death. In response to injury and inflammation, microglia and astrocytes could be activated immediately, or even overactive, which might be the potential reason for the occurrence of neurologic autoimmune disorders (Liddelow et al., 2020).

Nervous system autoantibodies mediate neurologic autoimmune disorders. According to the distribution of their target antigens, these nervous system autoantibodies could be roughly divided into PNS antibodies and CNS antibodies. In general, autoantibodies are stimulated by inflammation in specific organs and tissues. In some cases, cross-reactivity with microbial antigens could be detected. In specific conditions, such as virus infection, exposure to certain toxic chemicals, or neoplasms, antibodies targeting organs and tissue would trigger pathogenic status, which is always temporary when exposure is reduced or eliminated (Geng et al., 2020), resulting in the progression of autoimmune diseases. Autoantibodies, self-antigens, and other immune factors form an immune complex in the CNS and PNS, resulting in the dysfunction or destruction of neurons and glia (Kleopa, 2011).

In line with the clinical manifestations, the nervous system autoantibodies can be divided into: (1) autoimmune encephalitis-related antibodies: *N*-methyl-D-aspartate receptor (NMDAR), α-amino-3-hydroxy-5-metnyl-4-isoxazolepropionic acid receptor (AMPAR), gamma-aminobutyric acid B receptor (GABABR), voltage-gated potassium channel (VGKC), glycine receptor, metabotropic glutamate receptor 1 (mGluR1), metabotropic glutamate receptor 5 (mGluR5); (2) neuromyelitis pedigree-related antibodies: including myelin oligodendrocyte glycoprotein (MOG), myelin basic protein (MBP); (3) immune-mediated peripheral neuropathy-related antibodies: ganglioside M1 (GM1), ganglioside M2 (GM2), ganglioside M3 (GM3), ganglioside D1a (GD1a), ganglioside D1b (GD1b), ganglioside T1b (GT1b), ganglioside Q1B (GQ1b), myelin-associated glycoprotein (MAG), and thioester; and (4) immune-mediated neuromuscular junction disease-related antibodies: acetylcholine receptor (AChR) (Lalive, 2008; Zhang and Popovich, 2011; Gold et al., 2012; Pollak et al., 2016; Dalmau et al., 2017; Karim and Jacob, 2018). These autoantibodies not only attack neurons but also glial cells, leading to the imbalance of neural networks, disturbance of homeostasis, and inflammation hyperreaction, those further aggravate the destruction of neurons. In addition, the autoantibodies produced by autoimmunity or the autoantigens could recruit more glial cells in response to autoimmune reactions, resulting in neurologic autoimmune disorders in the nervous system. Autoantibodies targeting the aquaporin-4 (AQP4) channel, which is enriched on the surface of astrocytes and is involved in maintaining the stability of the blood–brain barrier (BBB), for example, cause neuromyelitis optica spectrum disorders (NMODSs).

Taken together, glial cells, as an important part of the nervous system, could be hyperactivated in the presence of autoantibodies or pathological changes, affecting the occurrence of neurologic autoimmune disorders. This review will focus on the roles of glial cells in neurologic autoimmune disorders and the influence of autoantibodies produced by autoimmune disorders on glial cells.

## Glial cells

As an important factor in the balance of the neural network, glial cells play a role in maintaining the stability and functional integrity of the nervous system (Casella et al., 2020; Healy et al., 2020). The types of glial cells mainly include astrocytes, oligodendrocytes, and microglia in the CNS. The Schwann cells and satellite glia are presented in the PNS.

### Glial cells in the central nervous system

Astrocytes are the most widely distributed glial cells in the CNS and have star-shaped cell bodies with numerous long and branching processes, which are important for supporting neurons through tight metabolic coupling (Ioannou et al., 2019). The fine morphology of the processes of astrocytes makes close contact with neuronal synapses, therefore, directly affecting local neurotransmission. Most importantly, astrocytes are critical for propagating inflammation, which contributes to disease development. On the other side, in response to immune reactions, as a crucial part of BBB, astrocytes prevent the inflammatory cells from entering the CNS during disease and injury (Heneka et al., 2018).

Oligodendrocytes are found throughout the gray and white matter in the CNS. In the adult brain, the cell body of oligodendrocytes is round or oval, with fewer protrusions wrapped around the neuronal axons and are responsible for the formation of myelin sheath in the CNS. Its function is crucial for forming an insulating cover around the axon, thus improving the transmission speed of electrical signals. Myelinating oligodendrocytes originate from oligodendrocyte progenitor cells (OPCs), which are maintained in the adult CNS. The abilities of proliferation and differentiation from OPCs into myelinating oligodendrocytes are critical for the progress of neurologic autoimmune disorders, for instance, MS (Falcao et al., 2018).

Microglia are the smallest glial cell type in the CNS. The cell body of microglia is small and short rod-shaped. The protrusions from the cell body are long and thin, and there are many small spinous processes on the surface. According to the function and status, microglia can be defined as three types, process-bearing, highly ramified myeloid cells, and tissue-resident macrophages (Greter et al., 2015). In response to injury and disorders, microglia are highly dynamic and activating (Nimmerjahn et al., 2005). Particularly in synapses of neurons, microglia are involved in complement-mediated synapse elimination, which is critical for neuronal circuit development (Schafer et al., 2012).

Interestingly, in the PNS, macrophages have similar functions as microglia in the CNS, for instance, presentation of antigens and cytokines production (Cao and He, 2013). Microglia have been shown to be derived from the embryonic yolk sac and migrate into the CNS (Ginhoux et al., 2010). Macrophages have two different origins, one part is from embryonic progenitors in the yolk sac and fetal liver, and another part is derived from hematopoietic stem cells (HSCs) in the bone marrow and blood monocytes (Crotti and Ransohoff, 2016). There is an overlap in the origin of microglia with that of tissue macrophages.

During inflammatory demyelination, macrophages from HSCs are thought to differentiate from two types of monocytes, the inflammatory monocytes, and the resident monocytes, and two groups exhibit important migratory and functional differences between mice and humans. Reports indicated that the inflammatory monocytes are pro-inflammatory and involved in demyelination in MS, whereas the resident group act as patrolling cells (Fogg et al., 2006).

### Glial cells in the peripheral nervous system

Schwann cells form the myelin sheath around the axons of neurons in the PNS. In the mature myelinated nerve axons, Schwann cells surround the segments between two Ranvier nodes of the nerve fibers, thus greatly accelerating the speed of neurotransmission in peripheral nerves. Therefore, when the myelin sheath of the PNS is damaged in neurologic autoimmune disorders, it ultimately affects the function of Schwann cells and axons. GBS is a rare neurologic autoimmune disease affecting the Schwann cells in the PNS (McGonigal et al., 2022).

Satellite cells surround the neuronal bodies of ganglion cells in the ganglia (such as sensory ganglia, sympathetic ganglia, and parasympathetic ganglia) of the PNS. Satellite cells have been found to play a variety of roles, including controlling the microenvironment of the sympathetic ganglia. They are considered to have similar functions to astrocytes in the central nervous system, providing support, nutrition, and protection for the surrounding neurons. In addition, satellite cells express a variety of receptors (such as purinergic receptors) that can interact with neuroactive substances (Kuang and Rudnicki, 2008).

## Neurologic autoimmune disorders and glial cells

### Multiple sclerosis

**FIGURE 1:**
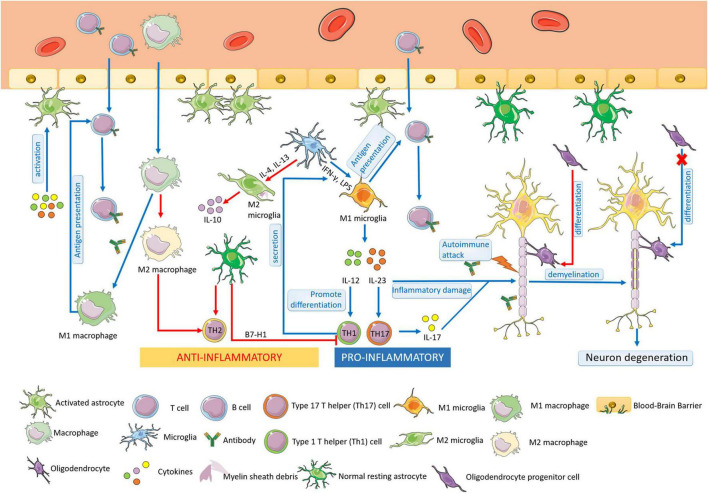
The pathogenesis of MS. Multiple sclerosis (MS) is an autoimmune disease characterized by chronic inflammation, demyelination, and gliosis in the CNS. At the early stage of MS, microglia could be activated and proliferated, which act as antigen-presenting cells. M1 microglia are differentiated by IFN-γ and LPS, secreting IL-12 and IL-23, promoting the differentiation of Th1 cells and Th 17 cells, leading to the aggravation of the inflammatory response of MS. M2 microglia are activated by IL-4/IL-13 and play a role in anti-inflammation. Macrophages have similar functions as microglia, including the presentation of antigens and cytokines production. M1 macrophages have the antigen-presenting ability the same as M1 microglia, resulting in the demyelination of nerves in MS. M2 macrophages increases Th2 cell differentiation to protect oligodendrocytes and neurons from damage. In MS, astrocytes can be activated, enhancing the permeability of BBB, and recruiting lymphocytes into the CNS. Moreover, astrocytes participate in the formation of BBB and secret immune inhibitory factors, acting in an anti-inflammatory role. The resting astrocytes inhibit the proliferation and promote apoptosis of Th1 cells and promote the secretion of anti-inflammatory cytokines by Th2 cells. Oligodendrocytes form the myelin sheath of CNS axons, providing nutrition and protecting nerve axons. At the early stage of MS, the regeneration and self-healing of nerve myelin sheath lesions are completed by the differentiation of OPCs into mature oligodendrocytes, but the differentiation ability is finally lost in progressive MS.

Multiple sclerosis is an autoimmune disease characterized by chronic inflammation, demyelination, and gliosis in the CNS. The most affected regions by MS in the CNS are the periventricular area, subcortical area, optic nerve, spinal cord, brainstem, and cerebellum (Filippi et al., 2018). The pathogenesis of MS is involved in the aberrant immune response directly against myelin antigen, such as the immune attack against MBP, resulting in the loss of myelin sheath in the white matter of the CNS. In the progress of MS, glial cells play an important role in the pathogenesis of MS autoimmunity (Figure 1). Pathological studies also suggested that the process of multiple demyelination in white matter is often accompanied by reactive gliosis (van Wageningen et al., 2022).

#### Microglia

Microglia have the functions of phagocytosis, antigen presentation, and cytokine production. At the early stage of MS, microglia could be activated and proliferated, which act as antigen-presenting cells. The activated microglia can be observed in the white matter of brain tissue in patients with early MS (Singh et al., 2013).

Studies show that microglia play a dual role in MS, which can not only promote an inflammatory response that aggravates tissue damage but also have neuroprotective and repair effects. The activated microglia could be divided into M1 and M2, which are attributed to their roles in MS, respectively. Cytokine interferon-γ (IFN-γ), lipopolysaccharide (LPS) induces microglia to differentiate into M1, which secretes interleukin-12 (IL-12) and interleukin-23 (IL-23). IL-12 is the main factor promoting the differentiation of type 1 T helper (Th1) cells; IL-23 promotes the type 17 T helper (Th17) cell differentiation, leading to the production of the inflammatory factor interleukin-17 (IL-17) secretion. Thus, the M1 microglia aggravate the inflammatory response of MS (Kong et al., 2016; Thompson and Tsirka, 2017). Microglia can be stimulated by interleukin-4 (IL-4)/ interleukin-13 (IL-13) to an M2 phenotype for the resolution of inflammation and tissue repair. In MS, M2 microglia have neuroprotective effects. The activation and proliferation of M2 microglia can remove extracellular oxidized proteins, phagocytic debris, and degenerated myelin sheath in brain tissue, provide a relatively suitable microenvironment for neuronal repair, and help to mend the damaged nervous system (Jackle et al., 2020).

#### Macrophages

Macrophages are as same important as microglia in the pathogenesis of MS. M1 macrophages have the antigen-presenting ability the same as M1 microglia, resulting in the demyelination of nerves in MS. Moreover, M2 macrophages play an anti-inflammatory role with microglia by increasing type 2 T helper (Th2) cell differentiation, therefore, protecting oligodendrocytes and neurons from damage (Cao and He, 2013).

#### Astrocytes

Astrocytes not only account for the majority of the CNS in quantity but also play a critical role in immune diseases of the CNS. Astrocytes can be activated by inflammatory factors, ischemia, hypoxia, and other stimuli. The permeability of BBB could be increased by activated astrocytes, facilitating the passage of lymphocytes to participate in the occurrence and development of lesions. Simultaneously, activated astrocytes directly or indirectly activate and recruit peripheral activated immune cells to the CNS by secreting cytokines, which is an important factor in the pathogenesis of MS (Farina et al., 2007; Aharoni et al., 2021). It was reported that the blockade of astrocyte CD38 activity suppressed pro-inflammatory transcriptional reprogramming, leading to the decreased expressions of pro-inflammatory transcriptional modules, which might contribute to CNS pathology in MS.

Moreover, astrocytes are not only involved in the occurrence of inflammation in the CNS but also act as important anti-inflammatory cells. At rest, as a maintainer of the stability of the CNS, astrocytes participate in the formation of BBB with vascular endothelial cells through its rich foot process structure, but also play a role in the biological barrier through the secretion of immune inhibitory factors. Studies have reported that normal resting astrocytes may inhibit the proliferation and promote apoptosis of T cells by expressing B7-H1 (Lipp et al., 2007). The proliferation of antigen-specific Th1 cells could be inhibited by astrocytes, which promote the secretion of anti-inflammatory cytokines by Th2 cells as well, thus inhibiting the development of inflammation (Liddelow et al., 2020). Therefore, the roles of astrocytes in the pathogenesis of MS still need further research.

#### Oligodendrocytes

Oligodendrocytes form the myelin sheath of CNS axons, providing nutrition and protecting nerve axons. As demonstrated, the pathological basis of MS is chronic inflammatory demyelination and neuronal degeneration of the CNS. At the early stage of MS, demyelinating lesions formed sclerotic plaques and had the function of nerve myelin regeneration and self-healing. However, with the development of MS, the functions of regeneration weaken and lose, eventually, the degeneration of nerve axons and neurons leads to brain parenchyma atrophy and severe disability (Brown et al., 2013). It is noted that the regeneration and self-healing of nerve myelin sheath at the early stage of MS lesions are completed by the differentiation of OPCs into mature glial cells (Meijer et al., 2022). Although OPCs always exist in the CNS, this differentiation ability in the lesions is finally lost in progressive MS (Ghorbani et al., 2022).

#### Clinical diagnosis and therapy

The pathology and the course of MS are sophisticated, suggesting that its clinical diagnosis and therapy might require targeting multiple biological processes. Although MS is generally considered to be triggered by autoimmune-mediated attacks of CNS myelin sheath, the anti-inflammatory treatments could not completely alleviate and prevent the development of MS, which is shown to be more and extensive axonal demyelination and degeneration (Trapp et al., 1998).

In response to neurologic autoimmune disorders, glial cells might be involved and subsequently connected to numerous biomarkers, which are critical and useful for diagnosis and therapy. In patients with MS, several myelin proteins including MBP and MOG have been detected and explored in cerebrospinal fluid (CSF) and serum, which are potential biomarkers for diagnosis.

Lenaldekar, an effective inhibitor of activated T cell proliferation, has been suggested to be a novel therapeutic approach for patients with MS. Moreover, in an experimental autoimmune encephalomyelitis (EAE) rodent model of MS, Lenaldekar treatment inhibited relapse severity, indicating a perspective application (Cusick et al., 2012). However, it is noted that no available animal model recapitulates the complicated and divergent pathogenesis of MS currently.

From the pathology of MS, preventing demyelination and promoting the regeneration of myelin sheath represent another potential therapeutic strategy for the treatment of MS. It is found that oligodendrocytes also produce nerve growth factor, neuregulin, glial-derived neurotrophic factor, and transforming growth factor (Acosta et al., 2013). These factors bind to corresponding receptors on neurons and glial cells and then affect the development and survival of neurons, astrocytes, and even oligodendrocytes themselves. In a word, oligodendrocytes play an important role in the progression and repair of MS disease (Zuchero and Barres, 2015). The enhancement of oligodendrocyte differentiation could be a way to help the regeneration of the myelin sheath (Rittchen et al., 2015); although many OPCs are in lesions sites at the early stage of MS, only rare maturing progenitors are found in chronic MS lesions, indicating that the differentiation capacity of OPCs is impaired in MS lesions (Kuhlmann et al., 2008).

### Guillain–Barre syndrome

Guillain–Barre syndrome is an acute immune-mediated polyneuropathy. Its pathophysiology can be divided into acute inflammatory demyelinating polyneuropathy (AIDP), acute sensory axonal neuropathy (ASAN), acute motor axonal neuropathy (AMAN), and acute motor and sensory axonal neuropathy (AMSAN). The main pathophysiological mechanism is complement-mediated nerve injury caused by antibody–antigen interaction in peripheral nerves. The anti-ganglioside antibody is the most common autoantibody in the development of GBS. GBS is induced by some immune trigger factors, most of which are precursory infections caused by pathogens, such as *Campylobacter jejuni*, *Haemophilus influenzae*, *Mycoplasma pneumoniae*, or *Cytomegalovirus*, and the current epidemic SARS-CoV-2 is also one of the infectious factors causing GBS (Kaida, 2019).

#### Schwann cells

In the PNS, axons are myelinated by Schwann cells. Gangliosides, a family of glycosphingolipids containing sialic acid, ensure the rapid transmission of myelinated axons by nerve fibers in the nervous system by regulating the caliber of axons and organizing ion channels in the Ranvier node and prevent axon regeneration after injury. The anti-ganglioside antibody can simulate this inhibitory effect on nerve repair (Lopez and Baez, 2018). Gangliosides GM1, GD1a, or GD1b are highly enriched at or near the Ranvier nodes (Kaida and Kusunoki, 2009) in axons myelinated by Schwann cells.

In human AMAN and AMSAN, early pathological features include the expansion of the Ranvier nodes, the deposition of complement products, and the complement system playing a central role in the immune response to eliminate invasive pathogens (Ramaglia et al., 2008). Therefore, in immune-mediated neuropathy associated with GM1, GD1a, or GD1b autoantibodies, complement-mediated Ranvier nodes destruction and activation of peripheral Schwann cells may be the common mechanism of these anti-ganglioside antibody-mediated neuropathies, explaining the continuous spectrum of AMAN, AMSAN, and ASAN (Susuki et al., 2012).

#### Other glia cells

Virus infection is also one of the main causes of GBS, and the effects of Zika virus and SARS-CoV-2 on glial cells in GBS are mainly discussed here. Some studies have shown that the Zika virus can replicate in microglia and cause cytopathy, resulting in a high degree of immune activation of microglia to resist pathogens in tissues. At the same time, it will also destroy normal tissues and cause neuropathy (Martinez Viedma and Pickett, 2018). Similarly, SARS-CoV-2 can also invade astrocytes, macrophages, and microglia in the CNS and induce the inflammatory response and increase the production of inflammatory mediators, including IL-1β and IL-2 (Raony et al., 2020). In addition, SARS-CoV-2 induces the expression of inflammatory cytokines in glial cells *in vitro*, such as IL-6, IL-12, IL-15, and TNF-α (Wu et al., 2020).

In conclusion, autoimmunity is the key to the pathophysiology of GBS. Glial cells present bacterial or viral particles to lymphocytes in the PNS, and the resulting immune response is accompanied by molecular simulation between pathogen epitopes and host gangliosides, such as *N*-acetylgalactosamine GD1a (GalNAc-GD1a), GD1a, GM1, ganglioside M1B (GM1b), and myelin proteins can lead to acute nerve injury and thus cause GBS (Mohammadi et al., 2020).

#### Clinical diagnosis and therapy

The progressive development of GBS is acute and serious, affecting the myelin sheath and the related axons. These damaged structures release biomarkers into the CSF. The candidate biomarkers for the diagnosis of GBS include MBP, axonal damage markers (neurofilaments, tau, anti-ganglioside antibodies), glial markers (S100B), and immunological markers (chemokines and complement factors) (Brettschneider et al., 2009). The applied potency of biomarkers in GBS is depending on several aspects: (1) the relevance to act as a surrogate for the disease development; (2) the practical results of prognostic accuracy; and (3) the potential to be predictors.

Intravenous immunoglobulin (IVIg) and plasma exchange have increasingly been used for the treatment of GBS (Yuki and Hartung, 2012). In addition, anti-GM1 IgG antibodies cause complement-mediated disruption of clusters of voltage-gated Na^+^ (Nav) channels at Ranvier nodes in peripheral motor nerve fibers of GBS animal models (Susuki et al., 2007). Application of complement inhibitors might reduce the formation of the membrane attack complex, prevent the disruption of sodium channel clusters, and lessen nerve injury in AMAN (Phongsisay et al., 2008).

### Neuromyelitis optica spectrum disorders

Neuromyelitis optica spectrum disorders are destructive inflammatory diseases of the CNS induced by autoantibodies, often causing demyelination of the CNS, mainly affecting the spinal cord, optic nerve, and brainstem. The typical manifestations are recurrent optic neuritis, longitudinal generalized myelitis, brainstem, diencephalon, and brain syndrome (Valencia-Sanchez and Wingerchuk, 2021). For many years, optic neuromyelitis has been considered a type of MS. Until 2004, researchers found that the main pathogenic autoantibody of NMODS in patients with optic neuromyelitis was AQP4 immunoglobulin G (AQP4-IgG) targeting AQP4 on the foot process membrane of astrocytes. For this reason, the disease is also known as CNS autoimmune astrocyte disease.

#### Astrocytes

AQP4 immunoglobulin G is a specific biomarker that can distinguish patients with NMODS from patients with MS. In addition, some patients with NMODS are AQP4-IgG seronegative. Therefore, the latest diagnostic criteria divide NMODS into AQP4-IgG seropositive and AQP4-IgG seronegative diseases (Paul et al., 2021). AQP4 is most strongly expressed in the CNS, mainly in astrocytes of brain, spinal cord, and optic nerve, especially on the pia mater and ependymal surface in contact with CSF (Rash et al., 1998). But it also exists in the epithelial cells of kidney (collecting duct), stomach (parietal cells), airway, gland, and skeletal muscle. It has been found that AQP4 is related to nerve excitation, astrocyte migration, and neuroinflammation, and the related contents have been summarized (Verkman, 2012).

In mice and *in vitro* experiments, the combination of AQP4-IgG and AQP4 activates complement-dependent cytotoxicity (CDC) and antibody-dependent cytotoxicity (ADCC) when killer cells are present. CDC may be the main mechanism of optic neuromyelitis. Some studies have also shown that the combination of AQP4-IgG and AQP4 can inhibit the aquaporin action or internalization of AQP4, because the loss of AQP4 expression in astrocytes can be observed in the early stage of NMODS (Carnero Contentti and Correale, 2021).

#### Other cells

After AQP4-IgG enters the CNS, it first causes astrocyte damage through CDC. A series of inflammatory reactions will then occur, leading to the infiltration of inflammatory cells, such as granulocytes, eosinophils, and lymphocytes, followed by the infiltration of macrophages and microglia. It can be detected that there is a high concentration of granulocyte colony-stimulating factors (Papadopoulos and Verkman, 2012) in the CNS at the time of the lesion. A large number of activated macrophages causes axonal damage by phagocytizing myelin and secreting pro-inflammatory cytokines, free radicals, glutamate, and metalloproteinases, resulting in a large number of deaths of oligodendrocytes, and finally leading to neuronal demyelination and necrosis (Hendriks et al., 2005).

#### Oligodendrocytes and patients with AQP4 immunoglobulin G negative

Aquaporin-4 is the first target antigen of autoimmune diseases of the CNS to be identified, but not all patients with NMODS have serum AQP4-IgG. The study found that 10–27% of patients with NMO had negative serum AQP4-IgG, but a large proportion (42%) of these patients with negative serum AQP4-IgG could detect MOG antibodies, especially in patients with recurrent optic neuritis (Hamid et al., 2017). MOG is a myelin protein located in the outermost layer of myelin sheath. Pathological studies of MOG-related diseases showed that there were well-defined areas of myelin loss in the lesion, and astrocytes and axons were relatively less damaged. The perivascular inflammatory infiltration was mainly CD4^+^ T cells and some B cells, and the deposition of complement could also be found. Due to different pathophysiology, serum biomarkers, clinical and radiological manifestations, and disease outcomes, MOG-related diseases are now regarded as unique disease entities (Paul et al., 2021).

In conclusion, the injury of astrocytes is the pathological basis of optic neuromyelitis, and then the infiltration of inflammatory cells and macrophages further leads to the necrosis of oligodendrocytes and finally the demyelination of neurons. This shows that demyelination is not the pathological feature of optic neuromyelitis, but a secondary phenomenon of astrocyte injury.

#### Clinical diagnosis and therapy

It has been demonstrated that up to several thousand times higher levels of glial fibrillary acidic protein (GFAP) could be detected in the CSF of patients suffering from NMODS compared to patients with MS (Takano et al., 2010). Therefore, except for AQP4-IgG, CSF GFAP could be a biomarker for examining astrocyte injury in patients.

### Myelin oligodendrocyte glycoprotein antibody-related diseases

#### Oligodendrocytes

Myelin oligodendrocyte glycoprotein is a highly conserved protein. It is only slightly expressed on the cell body of oligodendrocytes and the outer surface of the myelin sheath in the CNS (accounting for 0.05% of the total myelin protein). The exact role of MOG in the CNS remains unclear. By observing the extracellular domain of MOG, the researchers found that MOG exists in the form of dimer in natural or aqueous solution, so they speculated that MOG is an affinity adhesion receptor (Clements et al., 2003). In addition, MOG may also have the functions of stabilizing microtubules, activating complements, and participating in the transmission of information between cells (Johns and Bernard, 1999).

Myelin oligodendrocyte glycoprotein antibody-related disease (MOGAD) is a kind of autoimmune disease recently found (Dubey et al., 2019; Xie et al., 2022). The pathological manifestation of MOGAD is the demyelination of the CNS. Studies have shown that the most common clinical manifestation of MOGAD is optic neuromyelitis, in which AQP4-IgG serum negative optic neuromyelitis is the most common phenotype (Inan et al., 2020). Other clinical phenotypes include acute demyelinating encephalomyelitis and cortical encephalitis. Although there are overlapping clinical phenotypes between MOGAD, MS, and NMOSD, a large number of experiments and data show that MOGAD is a heterogeneous disease with characteristic clinical and imaging features (Shahriari et al., 2021).

The specific role of MOG antibodies (MOG-Abs) in the pathogenesis of MOGAD has not been clarified. At present, it is considered that most of the MOG-Abs are IgG I subtypes. By analyzing the complement activation in patients with MOGAD and healthy people, the researchers found that the proteins related to the classical complement activation pathway and alternative complement activation pathway increased significantly in patients with MOGAD (Keller et al., 2021), suggesting that complement activation is a prominent feature of MOGAD. The massive production of complement proteins will further activate CDC, resulting in extensive damage to oligodendrocytes in the CNS, which is manifested as neuronal demyelination. In addition to activating the complement pathway, denatured MOG protein can also activate T cell immunity (Ambrosius et al., 2020). The infiltration of inflammatory cells, the production of inflammatory factors, and the activation of macrophages will further aggravate the damaged myelinated axons by oligodendrocytes of the CNS.

In conclusion, oligodendrocyte injury leading to neuronal axon demyelination is the pathological basis and feature of MOGAD. The pathogenesis of MOGAD has not been fully clarified. It has been confirmed that the complement system will be activated at the onset, resulting in oligodendrocyte damage. At the same time, microglia and/or macrophages will also be activated to phagocytize myelin fragments and secrete inflammatory factors. The roles of other glial cells in the pathogenesis of MOGAD need further study.

### Peripheral neuropathy

Peripheral neuropathy is a kind of disease with the structure and dysfunction of motor sensation and autonomic nerve. Immune-mediated peripheral neuropathy is a neurological injury and dysfunction caused by the synergistic effect of humoral and cellular immunity. It has been found that the common mechanisms of several immune-mediated neurological diseases are as follows: (1) the body produces autoantibodies against myelin or protein in the Ranvier nodes; (2) the activated T cells can secrete chemokines and proteases, leading to the destruction of the blood–nerve barrier (Kieseier et al., 2018); and (3) macrophages break through the damaged blood–nerve barrier and destroy the nerve myelin sheath. The well-accepted hypothesis is the autoantibodies of nerve cell membrane and myelin. For example, injection of GM1 antibody into rabbits can cause a reversible intracellular block of the sciatic nerve (Pollard and Armati, 2011).

#### Schwann cells

Schwann cells are the main glial cells in the PNS, which are divided into myelinated Schwann cells and unmyelinated Schwann cells (Armati and Mathey, 2014). Schwann cells express many receptors, which recognize risk factors and activate local immune response by providing antigens and secreting cytokines and chemokines, attracting immune cells to the injury site. In order to avoid inflammatory overreaction, Schwann cells can also produce anti-inflammatory cytokine interleukin-10 (IL-10), which can inhibit the inflammatory response (Ydens et al., 2013).

#### Satellite cells

Satellite cells envelop sensory neurons in the ganglia, which suggest the communication between satellite cells and neurons through gap junctions without synaptic structures. Gap junctional coupling among satellite cells was found to be increased in pain models (Tracey, 2021). Interestingly, pain is a serious and common problem in patients suffering from MS, although the underlying mechanism is still unclear (Stenager et al., 1991). Even while MS has always been thought of as a CNS disease, research suggested that PNS may possibly be involved (Teixeira et al., 2020). Satellite cells activate and act through gap junctional coupling with neurons in the dorsal root ganglions (DRGs) in the peripheral component of pain syndrome in experimental MS animal models (Warwick et al., 2014). Further electrophysiological evidence also demonstrates that the membrane hyperexcitability of sensory neurons of DRG was found in animal models of MS, providing more clues for the sensitization of PNS in MS, but the mechanism and the roles of satellite cells in MS still need further investigations (Yousuf et al., 2019).

## Perspectives and conclusion

Although adaptive immunity to myelin antigens is essential in the pathogenesis of neurologic autoimmune disorders, innate immune mechanisms are likely involved in the initiation and perpetuation stages. Gut microbiota is closely related to the innate immune (Berer et al., 2011). Dysbiotic microbiota increases gut permeability and produces pro-inflammatory cytokines, stimulating inflammatory T cells, especially of the Th17 subtype, which is the same as that of myelin-destructive T cells. Therefore, T cells with cross-reactivity to myelin could become involved (Wekerle et al., 2013). In turn, circulating endotoxin may activate microglia, causing myelin sheath damage (Bergner et al., 2019). Therefore, the next question to ask is if there is a link between gut microbiota and neurologic autoimmune disorders (Zhang et al., 2020)? Studies in MS animal models suggest that the overactivated T cells are in line with the development of MS, which seems to require the presence of gut microbiota. It is reported that probiotic administration suppressed peripheral inflammatory responses in patients with MS (Tankou et al., 2018). Although a direct role for the gut immune system still needs to confirm, these clues for communication between gut microbiota and neurologic autoimmune disorders through glial cells suggest that the environmental factors might also be involved but needs further investigation (Hanninen, 2017).

To sum up, glial cells, as an important part of the nervous system, play an important role in the occurrence and development of autoimmune diseases in the nervous system. Glial cells are over-activated in the presence of autoantibody or pathology, altering the internal environment of neurons, releasing cytokines, and causing excessive synaptic pruning and extinction, resulting in neuroautoimmune diseases. In this process, autoantibodies are one of the important links. The autoantibodies produced by the body due to infection, environmental change, or virus invasion are often the cause of neurologic autoimmune disorders, and the injury of glial cells is also an important way to cause the disease. However, there are few studies on autoantibodies acting on glial cells, which need to be further explored and found. Second, the research on glial cells for neuroautoimmune diseases also suggests that we can treat such diseases through glial cells. For example, the use of anti AQP4 antibody can reduce the damage of astrocytes to neurons, to control the NMODS disease. We speculate the possibility of glial cells as a new direction for the study of neurologic autoimmune disorders, which provides a new idea for the potential clinical diagnosis and treatment.

## Author contributions

HL and Z-ZK designed and constructed the manuscript. Z-QL, T-XL, MT, Z-SR, C-YY, R-KY, and S-JS reviewed the literature and wrote the manuscript. All authors contributed to the article and approved the submitted version.

## Abbreviations

MS: multiple sclerosis
GBS: Guillain–Barre syndrome
NMODS: neuromyelitis optica spectrum disorders
CNS: central nervous system
PNS: peripheral nervous system
NMDAR: *N*-methyl -D-aspartate receptor
AMPAR: α -amino-3-hydroxy-5-metnyl-4-isoxazolepropionic acid receptor
GABABR: gamma-aminobutyric acid B receptor
VGKC: voltage-gated potassium channel
mGluR1: metabotropic glutamate receptor 1
mGluR5: metabotropic glutamate receptor 5
MOG: myelin oligodendrocyte glycoprotein
MBP: myelin basic protein
GM1: ganglioside M1
GM2: ganglioside M2
GM3: ganglioside M3
GD1a: ganglioside D1a
GD1b: ganglioside D1b
GT1b: ganglioside T1b
GQ1b: ganglioside Q1B
MAG: myelin-associated glycoprotein
AChR: acetylcholine receptor
AQP4: aquaporin-4
BBB: blood brain barrier
OPCs: oligodendrocyte progenitor cells
HSCs: hematopoietic stem cells
IFN-γ: cytokine interferon-γ
LPS: lipopolysaccharide
IL-12: interleukin-12
IL-23: interleukin-23
Th1: type 1 T helper
Th17: type 17 T helper
IL-17: interleukin-17
Th2: type 2 T helper
CSF: cerebrospinal fluid
AIDP: acute inflammatory demyelinating polyneuropathy
ASAN: acute sensory axonal neuropathy
AMAN: acute motor axonal neuropathy
AMSAN: acute motor and sensory axonal neuropathy
GalNAc-GD1a: *N*-acetylgalactosamine GD1a
GM1b: Ganglioside M1B
IVIg: intravenous immunoglobulin
Nav: voltage-gated Na^+^
CDC: complement-dependent cytotoxicity
ADCC: antibody-dependent cytotoxicity
GFAP: glial fibrillary acidic protein
MOGAD: myelin oligodendrocyte glycoprotein antibody-related disease
MOG-Abs: MOG antibodies
IL-10: interleukin-10
DRGs: dorsal root ganglions.
